# Identification of candidate genes for reproductive traits in Xinjiang sheep breeds based on genomic structural variation

**DOI:** 10.3389/fvets.2025.1551293

**Published:** 2025-06-06

**Authors:** Jiaqi Li, Sulaiman Yiming, Wenxin Zheng, Weiwei Wu, Jingdong Bi, Fengming Li, Mengting Zhu

**Affiliations:** ^1^College of Animal Science, Xinjiang Agricultural University, Urumqi, China; ^2^Institute of Animal Science, Xinjiang Academy of Animal Sciences, Urumqi, China

**Keywords:** sheep, whole-genome resequencing, structural variation, reproductive traits, selection signals

## Abstract

Reproductive traits are among the most important economic characteristics in sheep farming. Structural variations (SVs) are extensively distributed across sheep genomes and can directly or indirectly affect gene expression through a variety of mechanisms, leading to phenotypic variation among individuals or breeds. In this study, we characterized genomic SVs and identified candidate genes related to germplasm traits in seven sheep breeds. Based on the genome sequences of 73 ewes, SVs were detected using Delly, Lumpy, and Manta software tools, and the results were integrated using SURVIVOR software, leading to the identification of 107,166 SVs. The proportions of deletions, duplications, insertions, inversions, and translocations were 48.39, 38.41, 6.96, 6.22, and 0.03%, respectively. Regarding SV distribution, the number of SVs per chromosome decreased with increasing chromosome number. Both shared and breed-specific SVs were identified, with the local Tuva sheep breed showing the highest number of breed-specific SVs. Principal component analysis and phylogenetic tree results revealed a close genetic relationship between Hammari and Kabashi sheep. Selection signal analysis, gene annotation, and enrichment analysis led to the identification of potential functional SVs associated with reproductive traits, including *FSHR*, *ADCY5*, and *MTNR1A*. Experimental validation confirmed the regulatory activity of key SVs and their associations with the expression of target genes. This work characterized SVs in seven sheep breeds, identified genes associated with reproductive traits, and preliminarily validated the regulatory relationships between these SVs and their target genes. These findings provide essential data to support the genetic improvement of local sheep breeds in Xinjiang.

## 1 Introduction

Sheep (*Ovis aries*) are economically important animals in global livestock production. Reproductive efficiency is a key indicator of the income level in the livestock industry. Among the key economic traits, litter size is the most significant production characteristic, contributing 70 to 90% of the economic value in the sheep industry (1). The economic benefits of two-lamb litters are more than 1.6 times higher than those of single-lamb ones (2). Seasonal estrus is also a major factor limiting high productivity in sheep (3). Therefore, improving the reproductive capacity of sheep remains a key goal within the sheep industry, and is currently the subject of substantial research. Xinjiang possesses unique conditions for sheep farming and has cultivated many excellent sheep breeds with strong adaptability and disease resistance (4). For example, the local coarse wool breed, Kazakh sheep, is known for its resistance to cold, tolerance to coarse feed, strong adaptability, and good stress resistance. However, Kazakh sheep are seasonal breeders, typically producing one lamb per year, and the two-lamb rate is very low (5). Similarly, the newly developed Yunnan semi-fine wool sheep breed also exhibits seasonal breeding, with most births resulting in single lambs (6). Several foreign breeds, such as the Baikal sheep and Tuva sheep from Russia, and coarse wool breeds such as Kabashi and Hammari sheep from the Sudan, also commonly exhibit single-lamb births and seasonal breeding strategies (7–10). In contrast, the Hu sheep breed is known for its year-round estrus and, consequently, higher lambing rates (11).

Recent advancements in genomics have led to the development of new approaches to uncover the genetic basis of reproductive traits in sheep. Structural variants (SVs), major forms of genetic variation, have been shown to significantly influence important economic traits in livestock. Liang et al. (12) compared genomic SVs between Tibetan sheep, residing at high altitudes, and Hu sheep, typically found at low altitudes, seeking to determine how these variations impact gene expression during the high-altitude adaptation process in Tibetan sheep. They identified numerous SVs in the Tibetan sheep genome, which were widely distributed and had a significant impact on the expression of genes involved in key physiological processes, thus shedding light on the genetic mechanisms underlying high-altitude adaptation. Yang et al. (13) conducted a whole-genome analysis of SVs in sheep and goats and identified numerous SVs that emerged during their evolutionary history. These variations were found to be associated with key production traits and highlighted the convergent evolutionary features of sheep and goats during environmental adaptation. Notably, deletions (DELs) in genes such as *BMPR2* and *BMPR1B* were identified as critical regulators of litter size traits. Combining pan-genome construction, SV detection, and association analysis, Li et al. (14) explored the diversity and complexity of SVs in the sheep genome, and uncovered SVs showing significant associations with tail traits. Qiao et al. (15) employed whole-genome, long-read sequencing technology to systematically detect SVs across multiple sheep breeds. In addition to characterizing the types, sizes, and distribution patterns of these SVs, they also investigated their associations with genetic diversity among breeds. Their findings offer valuable resources and novel insights for genetic research, improvement, and molecular breeding in sheep.

However, research on the genetic basis of reproductive traits in sheep remains limited. In particular, there is a lack of systematic studies focusing on SVs. The aim of this study was to detect SVs in the genome sequences of 73 ewes across seven breeds and characterize these variations using Delly, Manta, and Lumpy software. High-frequency SVs were selected, annotated, and screened for candidate genes associated with reproductive traits. To further explore the function and role of these high-frequency SVs in reproductive traits, experimental validation was carried out. This study not only contributes to the understanding of the genetic mechanisms underlying reproductive traits in Kazakh sheep but also provides a theoretical framework for genetic research and improvements in molecular breeding in sheep.

## 2 Materials and methods

### 2.1 Data sources

This study employed whole-genome resequencing data from 73 ewes of seven sheep breeds. Blood samples (5 mL) were collected from six Kazakh sheep obtained from the Yili Kazakh sheep Farm into tubes containing EDTA anticoagulant, preserved on dry ice, and sent to the laboratory for DNA extraction. Whole-genome data for the remaining sheep [20 Yunnan semi-fine wool sheep (BioProject: PRJNA783661), 18 Hu sheep (BioProject: PRJNA1053506), 8 Kabashi sheep and 6 Hammari sheep from Sudan (BioProject: PRJNA849626), 4 Tuva sheep and 8 Baikal sheep from Russia (BioProject: PRJNA656153), and 3 Kazakh sheep (BioProject: PRJNA623062)] were obtained from the NCBI database.^1^

### 2.2 Detection of genomic structural variation

#### 2.2.1 DNA extraction and data processing

Genomic DNA was extracted from the samples using the phenol-chloroform method. The concentration and purity of the DNA samples were assessed using a NanoDrop 2000 Microvolume UV Spectrophotometer. Gel electrophoresis was employed to evaluate the integrity of the DNA samples, providing a comprehensive assessment of DNA quality. The DNA samples were stored at −80°C. Library construction and paired-end sequencing (Illumina HiSeq2500 platform; Illumina Inc.) were performed by Tianjin Compass Biotechnology Co., Ltd. (Tianjin, China).

#### 2.2.2 Quality control and alignment of genomic data

The raw data (FASTQ format) from paired-end sequencing were subjected to quality control using fastp software to obtain clean reads, which were then aligned to the sheep reference genome ARS-UI_Ramb_v3.0 using the BWA MEM algorithm (16). The alignment results were sorted and duplicates were removed using the SortSam and MarkDuplicates modules of PICARD software.^2^ Finally, the sorted BAM files were statistically analyzed using Qualimap software (17).

#### 2.2.3 SV detection and annotation

In this study, three software tools were employed for the detection and genotyping of SVs—Lumpy (18), Delly (19), and Manta (20). The results from Lumpy were further processed using SVTyper for SV genotyping. The results obtained via all three software tools were subsequently filtered and integrated using SURVIVOR (21), generating VCF files. The SVs were then annotated using SnpEff based on the annotation file of the sheep reference genome.

### 2.3 Population genetic analysis

#### 2.3.1 Principal component analysis

VCF files were converted to PLINK format using VCFtools v.0.1.17 and principal component analysis (PCA) was performed using PLINK (22) v.1.90. The results were visualized with the R package ggplot2.

#### 2.3.2 Phylogenetic tree construction

The phylogenetic tree was constructed using the neighbor-joining method. The Hamming distances between samples were calculated with PLINK software, generating a genetic distance matrix file. A Perl script was written to convert the file into .meg format, and the phylogenetic tree was then constructed using MEGA software. Finally, the graphical visualization of the tree was enhanced using the online tool ITOL.^3^

#### 2.3.3 Genetic differentiation index (*F*_ST_)

*F*_ST_ values for each sliding window were calculated using VCFtools, with a window size of 100 kb and a step size of 50 kb. The top 1% of these *F*_ST_ values were considered candidate regions under selection.

#### 2.3.4 Gene Ontology and Kyoto Encyclopedia of Genes and Genomes enrichment analysis of genes in selected regions

Gene Ontology (GO) and Kyoto Encyclopedia of Genes and Genomes (KEGG) enrichment analyses were conducted using DAVID^4^ and the results were visualized using the R package ggplot2.

### 2.4 SV validation

To validate the activity of RE-mediated SVs, dual-luciferase reporter gene vectors (pGL3-Basic vector) were constructed. Then, three SV sequences (FSHR-DEL, ADCY5-DEL, and MTNR1A-DEL) were separately cloned upstream of the reporter gene. SV activity was determined using the Luciferase Reporter Gene Assay Kit (Yeasen, Shanghai, China). Dual-luciferase activity was detected after 48 h post-transfection in HeLa cells.

## 3 Results

### 3.1 Sequencing, mapping, and SV detection

After quality control and alignment, the BAM files of several breeds were statistically analyzed using Qualimap software. A total of 17.43 GB of clean data were obtained. The alignment rate of clean reads from all the samples to the reference genome was 99.70%, with an average sequencing depth of 13.32×. The GC content was above 42.63% in all samples (Supplementary Table 1).

**Figure 1:**
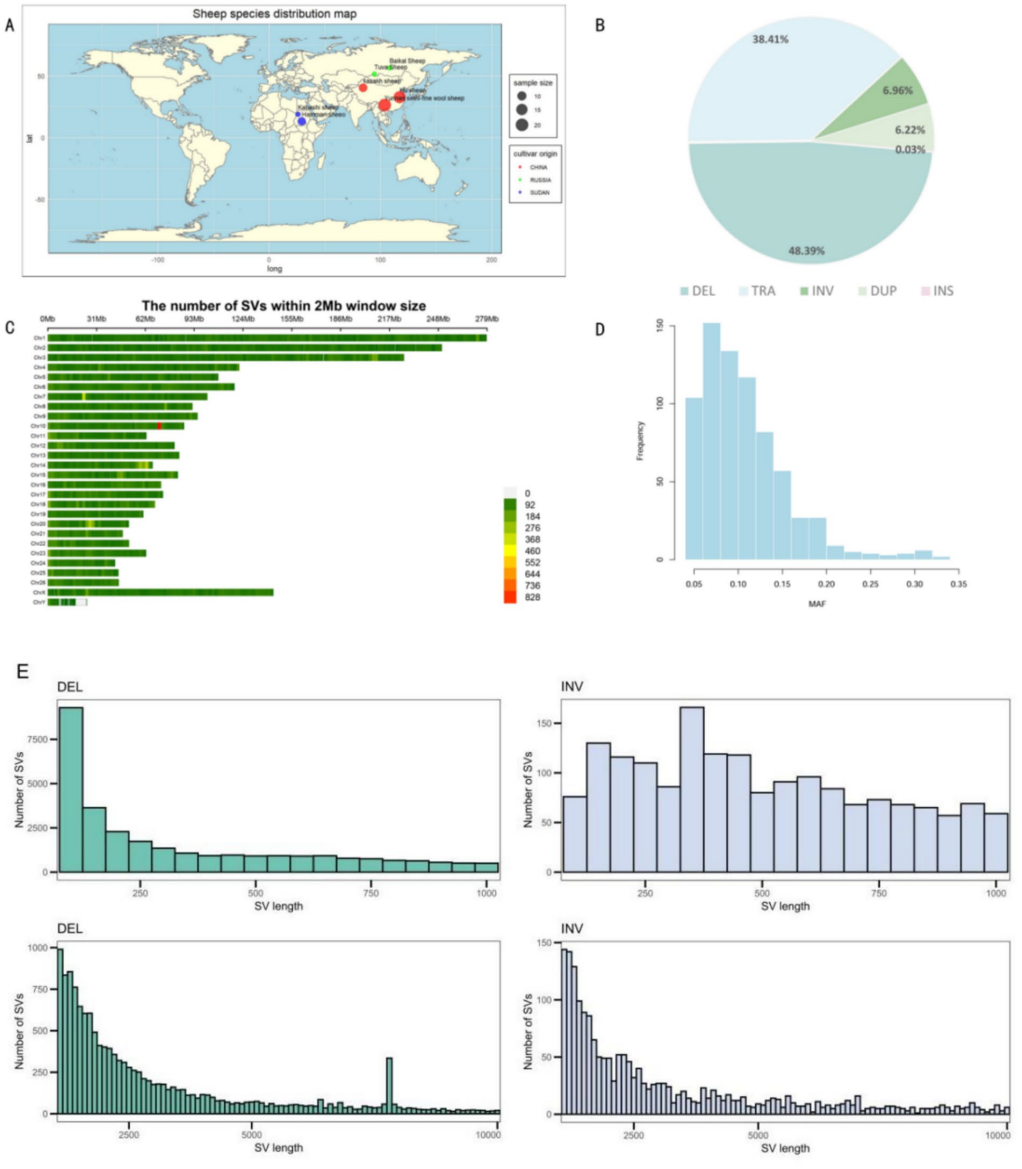
**(A)** Global geographic distribution of the seven sheep breeds. **(B)** Types of structural variants (SVs). **(C)** Chromosomal distribution of the SVs. **(D)** Minor allele frequency distribution across the genomes of 73 ewes. **(E)** Length distribution of the deletions and inversions.

Using Manta, Lumpy, and Delly software, a total of 107,166 SVs were identified from the seven sheep breeds from different regions (Figure 1A). Among the different types of variation, DELs were the most frequent, accounting for 48.39%, while insertions (INSs) were the least common, with only 27 SVs detected, representing 0.03% of the total. The numbers of duplications (DUPs) and inversions (INVs) were relatively similar (Figure 1B). Regarding their distribution, SVs were unevenly distributed across chromosomes, which was largely related to differences in chromosome length. The highest number of SVs was observed on chromosome 1, followed by chromosome 2. Notably, the number of SVs on the X chromosome was significantly higher than that on the Y chromosome (Figure 1C). Subsequently, minor allele frequencies were determined. The results showed that a large number of SVs were widely present in the sheep population, indicating that they are not rare (Figure 1D). Of the detected SVs, 21,335 DELs (35.11%) and 3,865 INSs (76.79%) had lengths ≥1 kb, and 3,800 DELs (14.38%) and 1,342 INSs (57.67%) had lengths ≥4 kb. Most DELs were in the 50 bp to 1 kb range, with their frequency significantly decreasing as their length increased (Figure 1E).

### 3.2 Distribution of SVs in different sheep breeds

**Figure 2:**
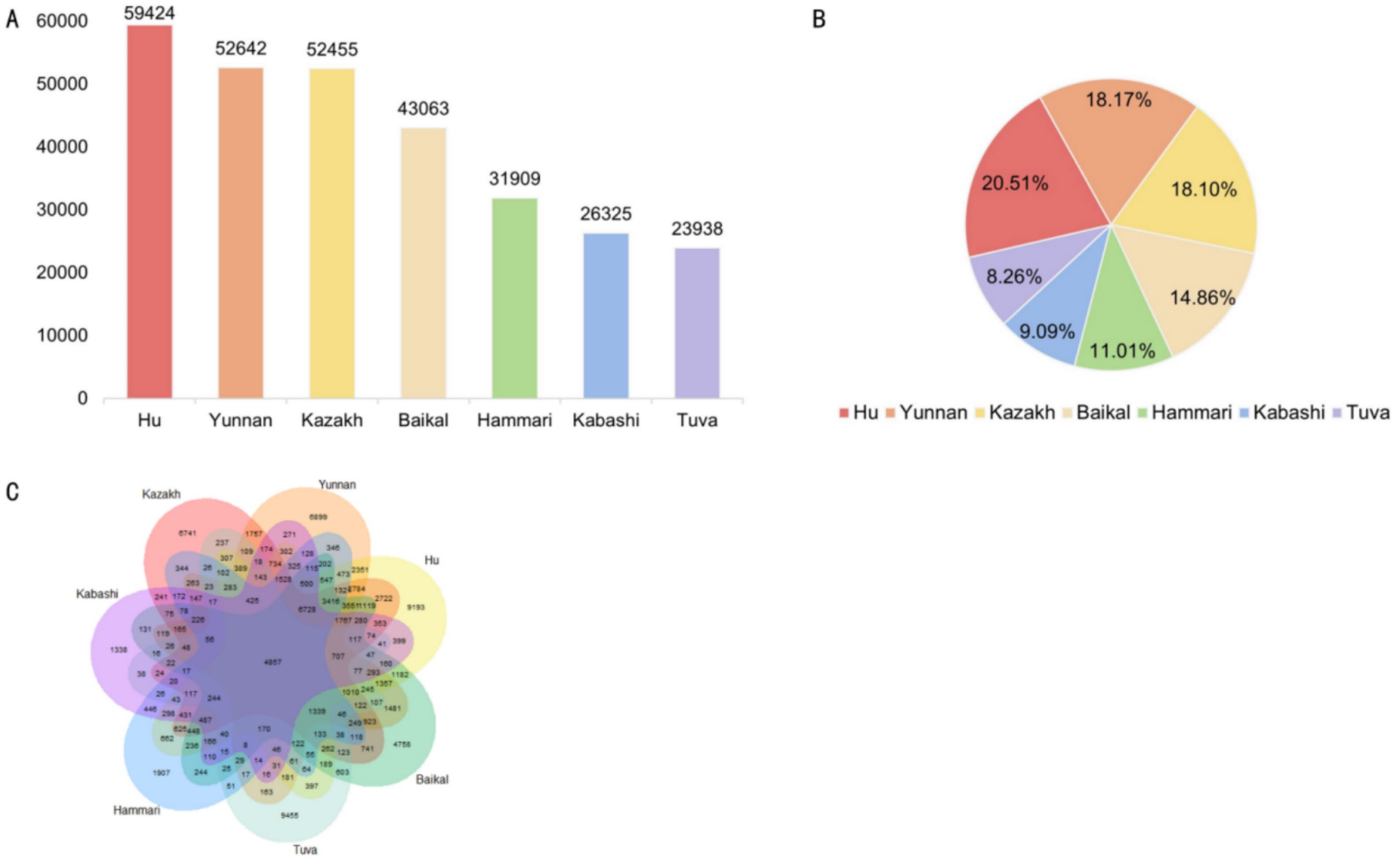
**(A)** The number of structural variants (SVs) in each breed. **(B)** The proportion of SVs in each breed. **(C)** A Venn diagram of the SVs in the seven breeds.

Across the seven sheep breeds, Hu sheep had the highest number of SVs, totaling 59,424, accounting for 20.51% of the total. The SV counts for Yunnan fine wool sheep, Kazakh sheep, Baikal sheep, Hammari sheep, Kabashi sheep, and Tuva sheep were 52,642, 52,455, 43,063, 31,909, 26,325, and 23,938, respectively (Figures 2A,B).

Further analysis showed that 4,857 SVs were shared among all seven breeds. The distribution of breed-specific SVs varied significantly, with Tuva sheep exhibiting the highest number of unique SVs (9,455), followed by Hu sheep (9,193), Yunnan semi-fine wool sheep (6,899), Kazakh sheep (6,741), Baikal sheep (4,758), Hammari sheep (1,907), and Kabashi sheep (1,338) (Figure 2C).

### 3.3 Population structure analysis

**Figure 3:**
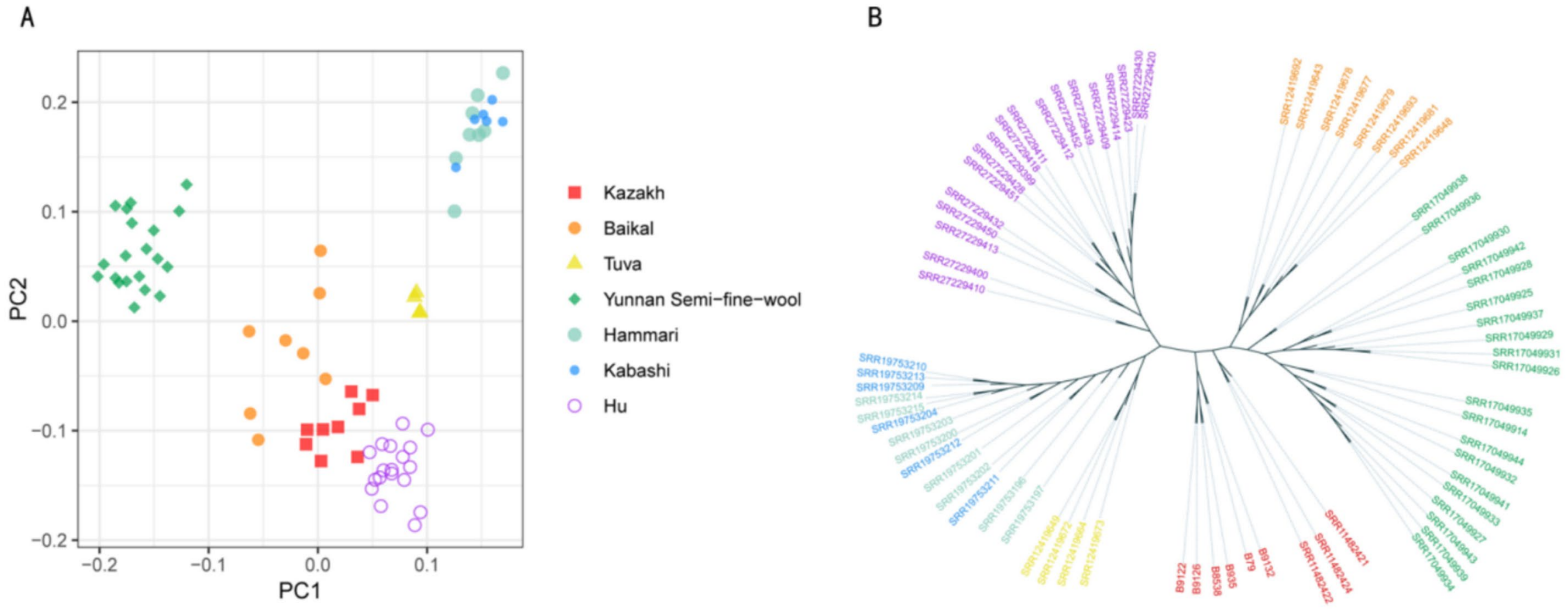
**(A)** Principal component analysis. **(B)** Phylogenetic tree analysis.

PCA of the SVs was used to explore the relationships among the breeds, plotting the first two principal components (PC1 and PC2). The analysis revealed that among the seven breeds, Hammari and Kabashi sheep clustered together, while the other five breeds showed clear separation based on their SV profiles (Figure 3A). To further investigate population relationships, a phylogenetic tree was constructed based on the SVs, with the results showing that the seven populations grouped into six distinct clusters, with Hammari and Kabashi sheep clustering together, consistent with the PCA results (Figure 3B). This was suggestive of a close genetic relationship between these two breeds.

### 3.4 Selection signal analysis

#### 3.4.1 Differences in SVs between Hu sheep and other breeds and between Hu sheep and Kazak sheep or Yunnan semi-fine wool sheep

**Figure 4:**
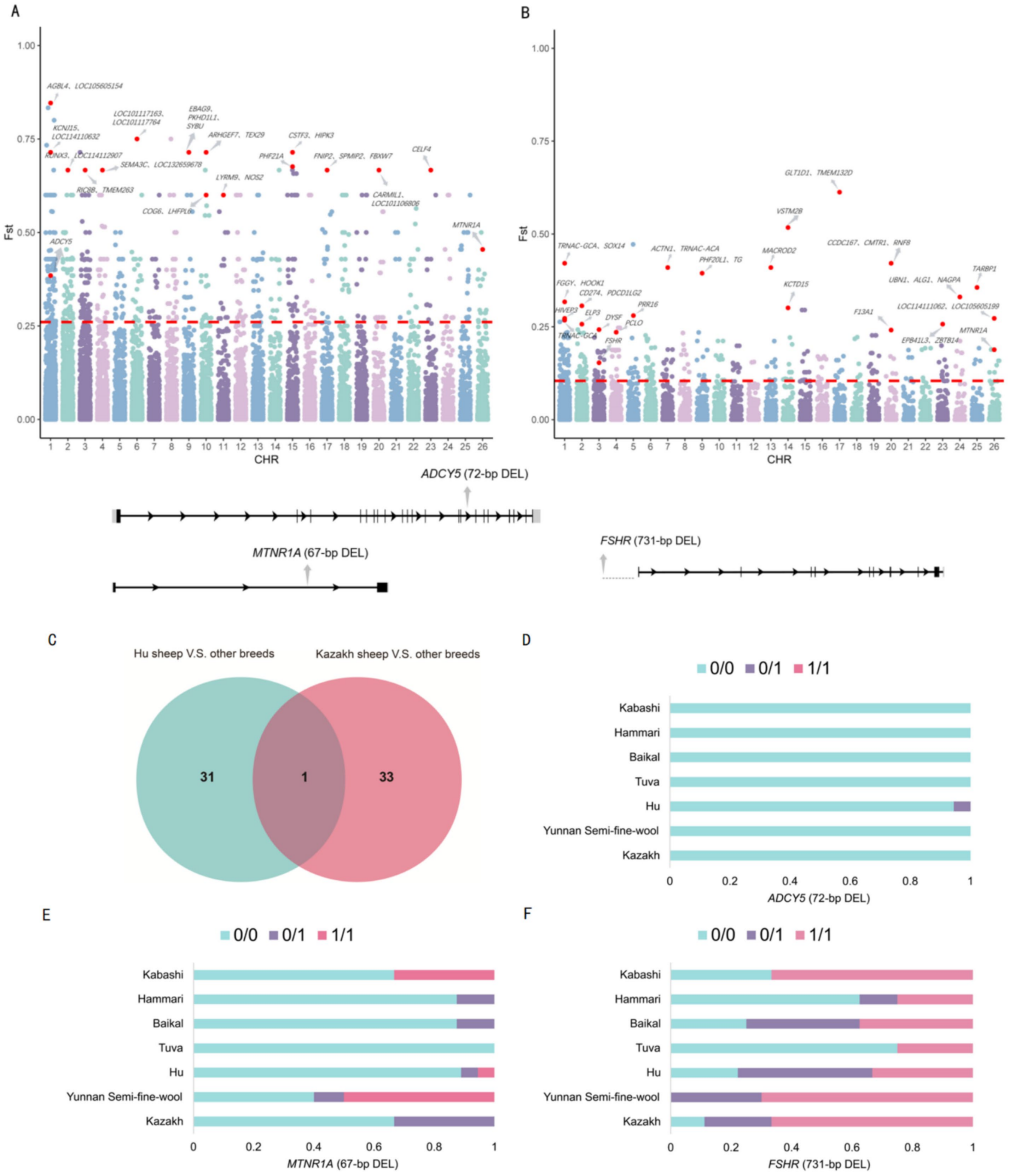
**(A)** A Manhattan plot for Hu sheep and the other breeds as well as between Hu sheep and Kazak sheep or Yunnan semi-fine wool sheep. **(B)** A Manhattan plot for Kazakh sheep and five other breeds. **(C)** A Venn diagram. **(D)** Genotype frequencies of the 72-bp deletion. **(E)** Genotype frequencies of the 67-bp deletion. **(F)** Genotype frequencies of the 731-bp deletion.

Selection signal analysis using *F*_ST_ detected SV differences between Hu sheep and other breeds, as well as between Hu sheep and Kazakh sheep or Yunnan semi-fine wool sheep. The top 1% of SVs with the highest *F*_ST_ values were selected as candidate regions for further analysis, leading to the identification of 653 loci under selection, annotated with 801 candidate genes. The *F*_ST_ threshold for the selected regions was 0.260 (Figure 4A).

#### 3.4.2 SV differences between Kazakh sheep and five other breeds

Next, we analyzed the differences in SVs between Kazakh sheep and five other breeds (Yunnan fine-wool sheep, Baikal sheep, Hammari sheep, Kabashi sheep, and Tuva sheep). The top 1% of SVs with the highest *F*_ST_ values were selected as candidate regions for further analysis, revealing that 358 loci were subject to selection, corresponding to 502 candidate genes. The *F*_ST_ threshold for the selected regions was 0.104 (Figure 4B).

#### 3.4.3 Shared and unique SVs detected

We identified a 67-bp deletion (*F*_ST_ = 0.188) in the intron of the *MTNR1A* gene, which was a shared high-*F*_ST_ SV between the two groups (Figure 4C). Additionally, a 72-bp deletion (*F*_ST_ = 0.385) in the intron of the *ADCY5* gene, which is associated with reproductive traits, showed high differentiation between Hu sheep and the other breeds, as well as among Hu sheep, Kazakh sheep, and Yunnan semi-fine wool sheep. Moreover, a 731-bp deletion (*F*_ST_ = 0.153) upstream of the *FSHR* gene, which is related to reproduction, was highly differentiated between Kazakh sheep and the other five breeds (Supplementary Table 1). Genotype frequencies for these three SVs were also statistically analyzed (Figures 4D–F).

### 3.5 Annotation and functional enrichment of candidate genes

**Figure 5:**
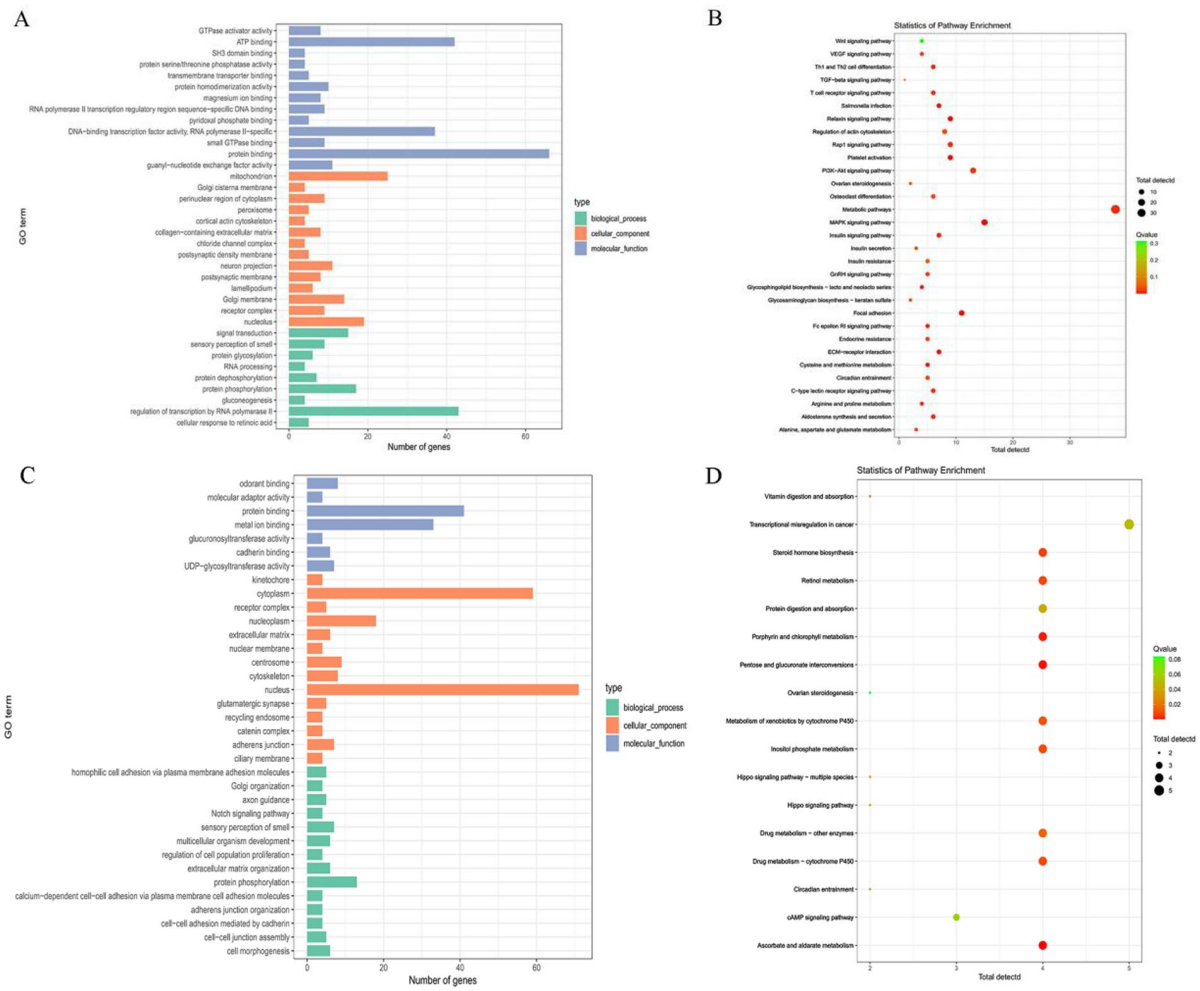
**(A,B)** GO functional enrichment analysis and KEGG pathway enrichment analysis of selected genes between Hu sheep and the other breeds, as well as between Hu sheep and Kazak sheep or Yunnan semi-fine wool sheep. **(C,D)** GO functional enrichment analysis and KEGG pathway enrichment analysis of selected genes between Kazakh sheep and the other five breeds.

GO term and KEGG pathway enrichment analyses were performed on the selected regions using the DAVID and KOBAS websites. GO enrichment analysis between Hu sheep and the other breeds, as well as between Hu sheep and Kazakh sheep or Yunnan semi-fine wool sheep, identified 36 GO terms, mainly related to processes such as protein binding, ATP binding, mitochondrion, and regulation of transcription by RNA polymerase II (Figure 5A). Meanwhile, KEGG pathway enrichment analysis revealed 31 pathways, primarily associated with the WNT signaling pathway, Relaxin signaling pathway, GnRH signaling pathway, and ovarian steroidogenesis (Figure 5B). GO enrichment analysis between Kazakh sheep and the other five breeds identified 35 GO terms, mainly linked to processes such as protein binding, metal ion binding, nucleus, and protein phosphorylation (Figure 5C). Finally, KEGG pathway enrichment analysis revealed 17 pathways, mainly related to ovarian steroidogenesis, steroid hormone biosynthesis, Hippo signaling pathway, and circadian entrainment (Figure 5D).

### 3.6 Functional validation of high-frequency SVs associated with reproductive traits

**Figure 6:**
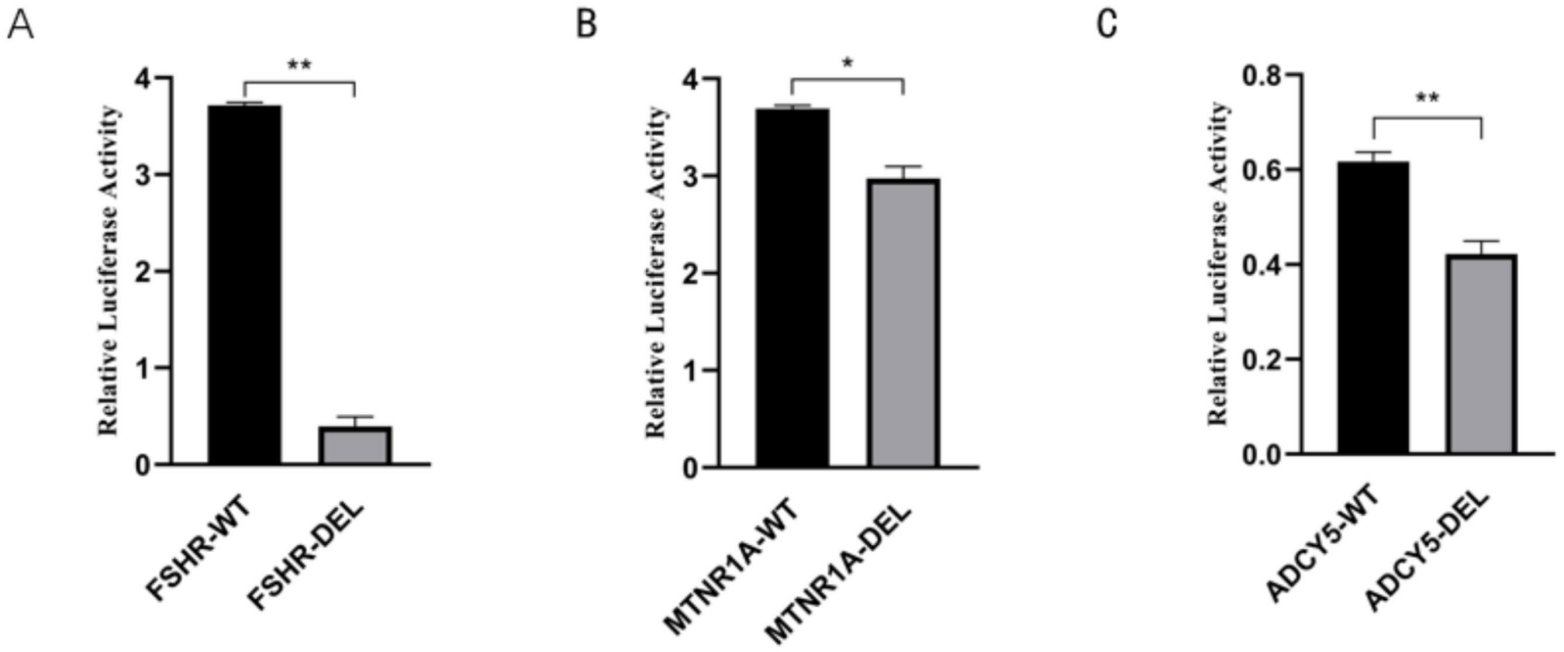
**(A)** Dual-luciferase reporter assay of the 731-bp sequence in HeLa cells. **(B)** Dual-luciferase reporter assay of the 67-bp sequence in HeLa cells. **(C)** Dual-luciferase reporter assay of the 72-bp sequence in HeLa cells.

We identified three high-frequency SVs associated with reproductive traits, including a 731-bp DEL upstream of the *FSHR* gene. This variant was found to be most prevalent in the Yunnan fine wool sheep population (AF = 0.70), followed by the Kazakh (AF = 0.68) and Kabashi (AF = 0.68) sheep populations. The frequencies were lowest in the Tuva sheep (AF = 0.25) and Hammari sheep (AF = 0.25) populations. Dual-luciferase reporter assays demonstrated that the 731-bp DEL led to a reduction in enhancer activity, with significantly higher luciferase expression observed in cells carrying the *FSHR*-DEL variant than in those with the *FSHR*-WT genotype, suggesting that the 731-bp sequence has enhancer activity and promotes *FSHR* gene expression (Figure 6A). Similarly, our findings revealed that both the 67-bp DEL in the intron of the *MTNR1A* gene and the 72-bp DEL in the intron of the *ADCY5* gene resulted in a significant reduction in luciferase expression in cells with the *MTNR1A*-DEL and the *ADCY5*-DEL compared to that in WT cells (Figures 6B,C). These results indicated that both DNA fragments act as enhancers in HeLa cells.

## 4 Discussion

SVs represent an important source of genetic diversity and have been extensively studied in plants and animals over recent years (23). In this study, we used three software tools—Manta, Lumpy, and Delly—to detect SVs and then generated a SV set by considering only those detected by all three tools. This approach aimed to improve detection accuracy and minimize false positives. A total of 107,166 SVs were identified. In terms of variation types and quantities, DELs accounted for the highest proportion at 48.39%, while TRAs represented only 0.03%. We detected genomic SVs in seven sheep breeds, with the highest number found in Hu sheep and the lowest in Tuva sheep. However, when breed-specific variations were considered, Tuva sheep exhibited the highest number of unique variations, followed by Hu sheep. This high number of unique SVs in Tuva sheep, a local breed with a unique genetic background, likely reflects the accumulation of a large number of breed-specific variations due to long-term natural selection and genetic drift. A Venn diagram analysis indicated that 4,857 SVs were shared among the seven breeds, with additional shared SVs detected between specific breeds. Due to the limitations of currently available methods for the detection of INSs, it is necessary to combine long-read sequencing and third-generation sequencing to improve SV detection in the future.

PCA was performed to assess the genetic relationships among the seven sheep breeds. The results showed that, except for Hammari and Kabashi sheep, which clustered together, the other five breeds exhibited significant genetic differentiation. Further analysis based on phylogeny confirmed the PCA results, with the seven populations grouped into six branches. Hammari and Kabashi sheep were again placed in the same branch, suggestive of a high degree of genetic similarity between the two. The consistency between the PCA and phylogenetic tree results suggests that Hammari and Kabashi sheep may share a closer evolutionary origin or experience more frequent gene flow. This genetic relationship may be linked to their geographic distribution, historical breeding, or ecological adaptations. Moreover, the significant genetic differentiation observed among the other five breeds likely reflects breed-specific selective pressures and the long-term environmental effects on these populations.

In this study, we identified several candidate genes related to reproductive traits, including *MTNR1A*, *FSHR*, and *ADCY5*. Previously, Lukic et al. (24) identified the *MTNR1A* gene as playing a significant role in regulating seasonal reproduction in sheep using a genome-wide analysis. The *MTNR1A* gene, which encodes the main receptor for melatonin, regulates melatonin signaling, influencing the activity of the hypothalamic-pituitary-gonadal axis in sheep. Consequently, *MTNR1A* regulates the reproductive cycle and performance of these animals and enables them to better adapt to seasonal environmental changes. Arjoune et al. (25) used genotyping and statistical analysis to explore the genetic polymorphisms of the *MTNR1A* gene in two Mediterranean sheep breeds and examined how these polymorphisms affect reproductive performance. The authors reported that certain *MTNR1A* genotypes were significantly associated with favorable reproductive traits, such as higher lambing rates, better fertility, and more stable estrous cycles. Abuzahra et al. (26) summarized existing studies on the relationship between *MTNR1A* and lambing rates in sheep, reviewing the role of the *MTNR1A* gene in reproductive performance, particularly lambing rates, and analyzed how its polymorphisms influence reproductive capacity. Their findings suggested that *MTNR1A* plays a crucial role in regulating sheep reproductive cycles and efficiency. Employing whole-genome sequencing, Guo et al. (27) found that the *FSHR* gene is closely associated with reproductive traits (e.g., parity) in sheep and plays a significant role in selective breeding. Ma et al. (28), using specific locus amplified fragment sequencing (SLAF-seq), identified novel genes associated with lambing rates in Xinjiang sheep populations, including *FSHR*, which influence reproductive performance by regulating reproductive processes. He et al. (29) demonstrated through qRT-PCR that the expression level of the *FSHR* gene was significantly higher in high-reproduction sheep than in low-reproduction ones and identified several polymorphic sites in this gene that were closely related to reproductive performance. Similarly, Tao et al. (30) found a significant correlation between SNPs in the *FSHR* gene and reproductive traits in different sheep breeds using genotyping, qPCR, and bioinformatics analysis. Pan et al. (31) further characterized the *FSHR* gene, analyzed its expression patterns across various tissues and conditions, and systematically explored the potential association between *FSHR* polymorphisms and lambing rates. They concluded that the expression characteristics and genetic variations of the *FSHR* gene significantly influenced reproductive performance in sheep. Finally, Du et al. (32), using comparative transcriptomic analysis, identified a seasonal expression pattern of the *ADCY5* gene in the adrenal tissues of Sunit sheep, confirming it as a key candidate gene influencing seasonal reproduction.

In this study, we identified 107,166 genomic SVs, greatly expanding our understanding of the genetic variation across different sheep breeds. The identified high-frequency SVs associated with reproductive traits offer valuable insights into potential candidate genes for use in Xinjiang native sheep breeding. Overall, this study provides a valuable genetic resource for understanding sheep domestication and reproductive traits and contributes to the dissection of the genetic basis of important phenotypic traits.

## Data Availability

The datasets presented in this study can be found in online repositories. The names of the repository/repositories and accession number(s) can be found in the article/Supplementary material.

## Footnotes

^1^ https://www.ncbi.nlm.nih.gov/sra/

^2^ https://broadinstitute.github.io/picard/

^3^ https://itol.embl.de/

^4^ https://david.ncifcrf.gov/

## Supplementary material

SUPPLEMENTARY FIGURE S1 FSHR fluorescence transfection efficiency.

SUPPLEMENTARY FIGURE S2 ADCY5 fluorescence transfection efficiency.

SUPPLEMENTARY FIGURE S3 MTNR1A fluorescence transfection efficiency.
