# Supplementary figures and images for: Identification of candidate genes for reproductive traits in Xinjiang sheep breeds based on genomic structural variation
Source: Front Vet Sci. 2025 Jun 6;12:1551293. doi: 10.3389/fvets.2025.1551293 (PMC12184774; doi:10.3389/fvets.2025.1551293)

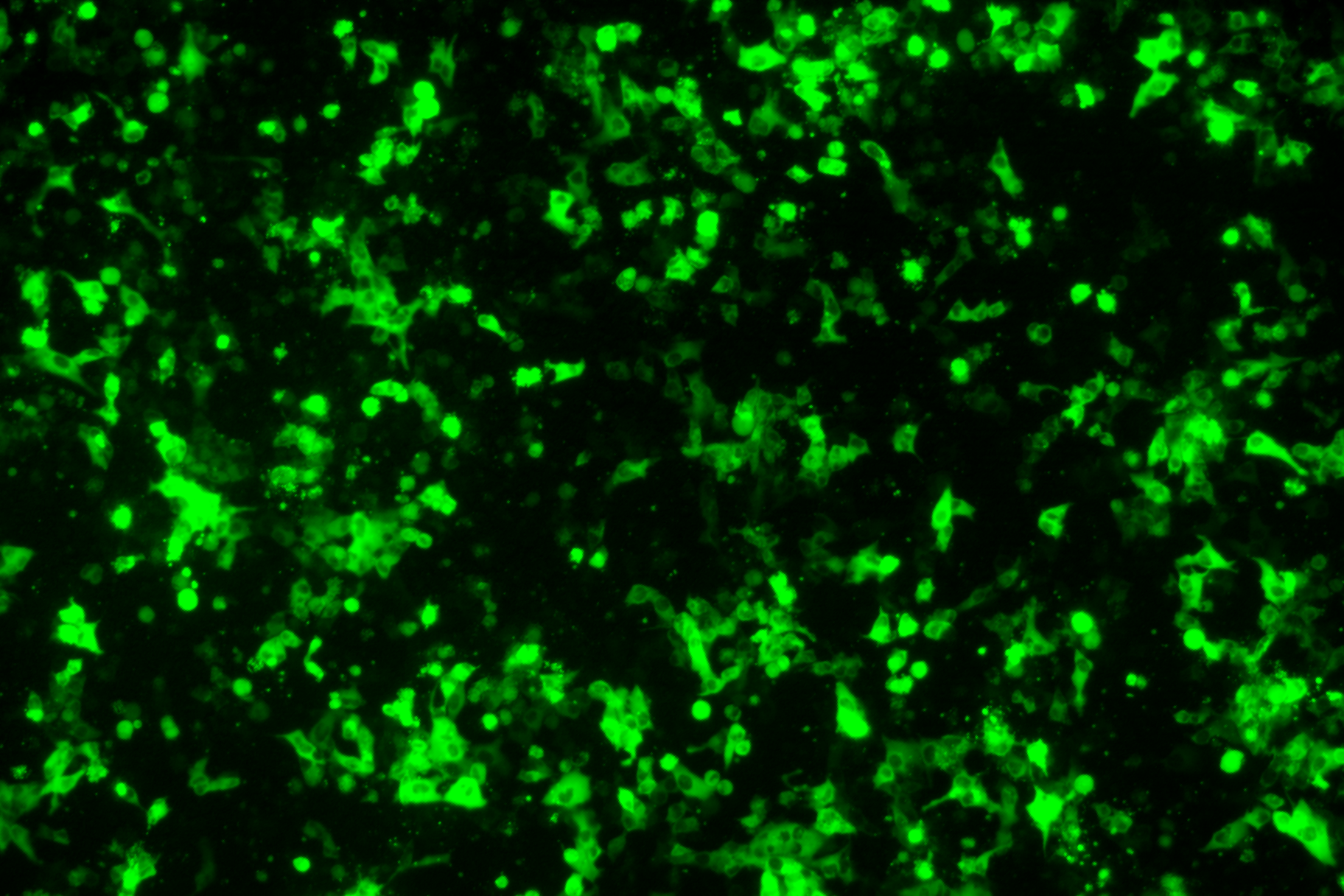

Supplement: SUPPLEMENTARY FIGURE S1 — FSHR fluorescence transfection efficiency. [file Image_1.tif]

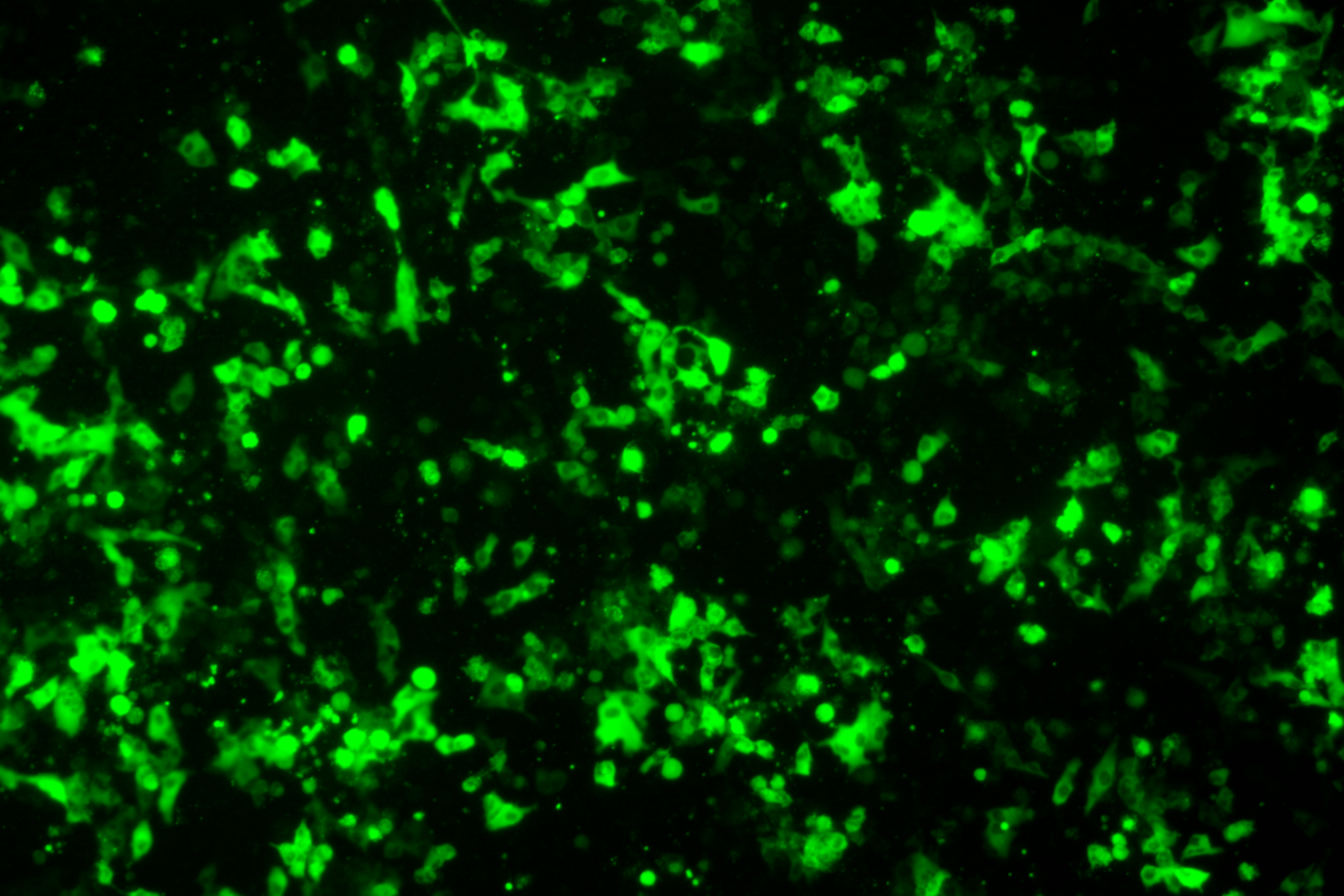

Supplement: SUPPLEMENTARY FIGURE S2 — ADCY5 fluorescence transfection efficiency. [file Image_2.tif]

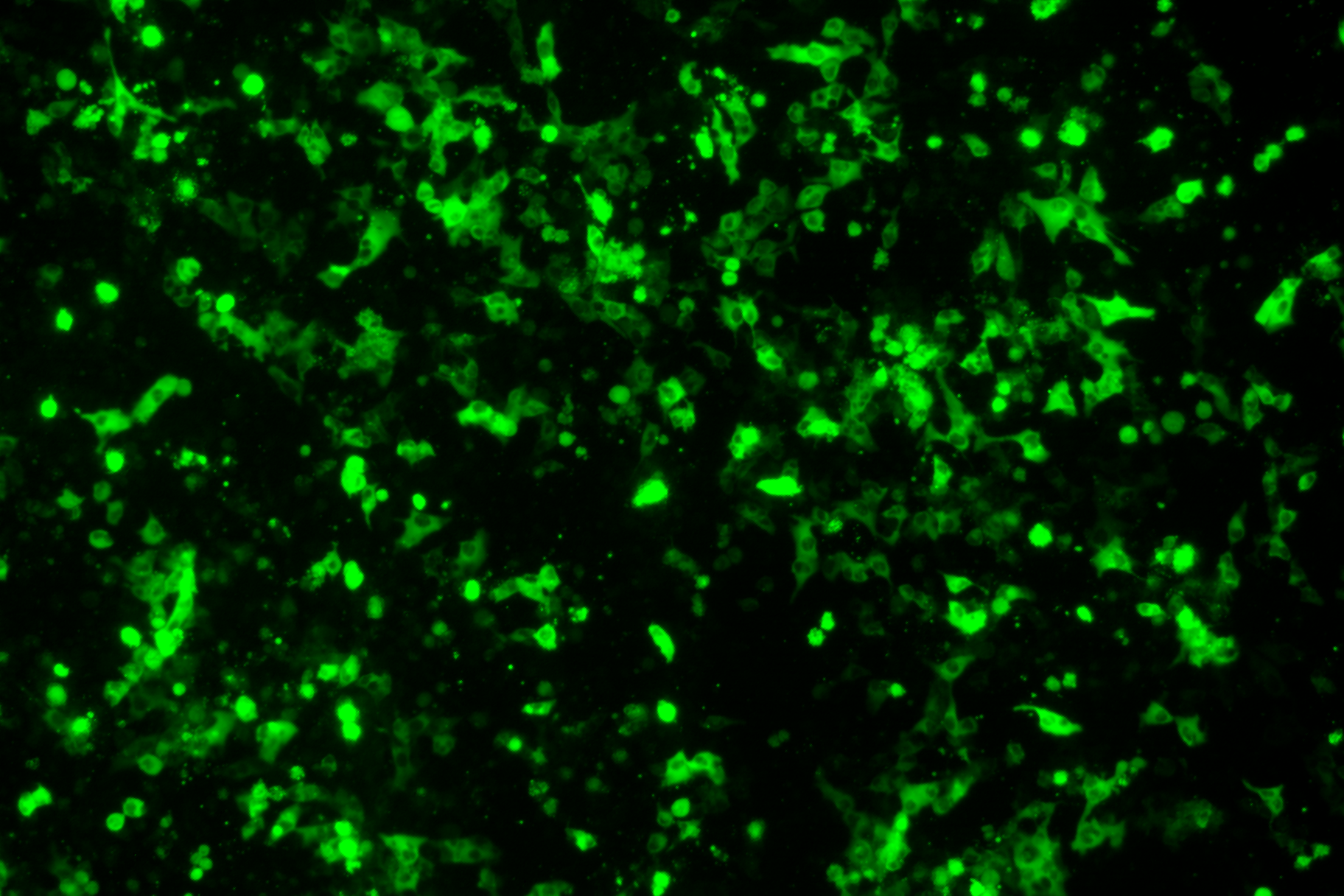

Supplement: SUPPLEMENTARY FIGURE S3 — MTNR1A fluorescence transfection efficiency. [file Image_3.tif]
